# *Helicobacter pylori* causes gastric dysbacteriosis in chronic gastritis patients

**DOI:** 10.1515/biol-2022-0839

**Published:** 2024-03-28

**Authors:** Chao Cen, Qiuying Du, Bin Luo, Tonghua Wang, Jianwei Su, Xiaoshan Qin, Wenyan Zhang, Lijing Lu, Yang Liao, Yanqiang Huang, Yumei Liang

**Affiliations:** Department of Gastroenterology, Affiliated Hospital of Youjiang Medical University for Nationalities, Baise, Guangxi 533000, China; Graduate School of Youjiang Medical University for Nationalities, Baise, Guangxi 533000, China; Department of Laboratory, Affiliated Hospital of Youjiang Medical University for Nationalities, Baise, Guangxi 533000, China; Department of Pathogen Biology and Immunology, Youjiang Medical University for Nationalities School of Basic Medical Sciences, Baise, Guangxi 533000, China; Department of Neonatology, Affiliated Hospital of Youjiang Medical University for Nationalities, No. 18, Zhongshan Second Road, Youjiang District, Baise, Guangxi 533000, China

**Keywords:** chronic gastritis, *Helicobacter pylori*, 16S ribosomal RNA, operational taxonomic unit, Kyoto encyclopedia of genes and genomes

## Abstract

Gastric mucosal samples were procured and underwent the sequencing of 16S ribosomal RNA (16S rRNA) via Illumina high-throughput sequencing technology to explore the impact of *Helicobacter pylori* (*H. pylori*) infection on the composition of gastric flora in chronic gastritis (CG) patients. In the results, the operational taxonomic unit (OTU) analysis revealed an overlap of 5706 OTUs shared between the two groups. The top 5 abundance ranking (TOP5) phyla comprised Bacteroidetes, Proteobacteria, Firmicutes, Actinobacteria, and Epsilonbacteraeota, while the TOP5 genus was *Lachnospiraceae_NK4A136_group*, *Helicobacter*, *Bacteroides*, *Klebsiella*, and *Pseudomonas*. In the metabolic pathways at the Kyoto Encyclopedia of Genes and Genomes (KEGG)_L3 level, conspicuous variations across seven functions were observed between the *H. pylori*-positive (HP_Pos) and *H. pylori*-negative (HP_Neg) groups. Subsequently, functional gene enrichment in KEGG pathways was further validated through animal experimentation. In contrast to the mice in the HP_Neg group, those infected with *H. pylori* manifested an infiltration of inflammatory cells, an augmentation in gastric acid secretion, and conspicuously elevated scores regarding gastric activity, along with heightened levels of malondialdehyde. In conclusion, CG patients infected with *H. pylori* displayed a disorder in gastric flora, furnishing a theoretical basis for the prophylaxis of *H. pylori* infection and its associated pathogenic ramifications.

## 1 Introduction

Chronic gastritis (CG) manifests as a malady characterized by persistent inflammatory or atrophic lesions within the gastric mucosa, holding a preeminent position in terms of prevalence among various gastric disorders [1]. The principal prototypes of CG include chronic superficial gastritis (CSG), chronic erosive gastritis, and chronic atrophic gastritis (CAG). Amidst the phase of CAG, patients become notably susceptible to gastric cancer, a reality acknowledged by the World Health Organization’s classification of CAG as a precancerous state in the evolution of gastric cancer [2]. Nonetheless, the majority of these alterations can be reversed during the CSG phase, thereby emphasizing the critical importance of prompt and efficacious management of CG.

The onset of CG is initiated by diverse etiological factors, encompassing elements such as *Helicobacter pylori* (*H. pylori*) infection [3], autoimmunity [4], and pharmaceutical influences [5]. Of these, *H. pylori* infection constitutes approximately 90%, emerging as the predominant etiology of CG [6]. *H. pylori*, a spiral-shaped Gram-negative bacterium, was initially isolated from the gastric mucosa of patients with CG in the 1980s and cultivated in a microaerobic environment, indicating a robust association between *H. pylori* and CG [7]. Upon adhering to the gastric mucosa, *H. pylori* injects virulence factors into the gastric epithelial cells, inciting chronic inflammatory and immune responses, which gradually inflicts damage upon the gastric mucosa and further exacerbates gastric tissue damage [8]. Nevertheless, the precise pathogenic mechanism through which *H. pylori* infection gives rise to CG remains ambiguous, warranting further in-depth investigation.

In recent years, the cultivation and high-throughput sequencing of gastric juice and biopsies have unveiled the intricate bacterial microbiota inhabiting the human stomach [9]. The gastric microorganisms predominantly encompass Proteobacteria, Phylum Firmicutes, Bacteroidetes, and Actinobacteria, assuming crucial roles in digestion, absorption, metabolism, immunity, and the restraint of pathogen colonization [10]. They maintain the ecological equilibrium in the stomach, and any structural or functional imbalance among them has the potential to precipitate various maladies [11]. The diversity of microorganisms varies with the health status of the gastric mucosal epithelium, with an inverse correlation manifesting between *H. pylori* and the diversity of the gastric microbiota [12]. The colonization of *H. pylori* results in a reduction in bacterial diversity, profoundly disrupting the microecological environment in the stomach, thus leading to various gastric maladies and ultimately impacting the health of the host [13]. Nevertheless, the extent to which *H. pylori*, a pivotal pathogenic bacterium in CG, is associated with the alterations in the gastric microbiota of CG patients remains a domain that has not yet undergone comprehensive exploration.

In this investigation, specimens were procured from the gastric mucosa of *H. pylori*-positive (HP_Pos) and *H. pylori*-negative (HP_Neg) CG patients. The differences in bacterial communities were examined employing 16S ribosomal RNA (16S rRNA) sequencing technology to delve into the impact of *H. pylori* infection on the composition of the gastric flora. Additionally, this study unveiled the underlying pathogenic mechanism of *H. pylori* infection through the further animal experiments. Such revelations establish a theoretical basis for the subsequent endeavor of pursuing novel remedies targeting the restoration of the microecological balance of gastric mucosa, as well as for the prevention of *H. pylori* infection and its associated pathogenic ramifications.

## 2 Materials and methods

### 2.1 Patients and samples

This study investigated and screened both outpatients and inpatients exhibiting upper gastrointestinal symptoms who underwent gastroscopy at the Endoscope Center of the Affiliated Hospital of Youjiang Medical University for Nationalities between June 2020 and November 2020. This study completed registration in the Chinese clinical trial registry (registration website: https://www.chictr.org.cn/index.html). The research received approval from the Medical Ethics Review Committee of the Affiliated Hospital of Youjiang Medical University for Nationalities (approval number: YYFY-LL-2017-01), and the written informed consent was obtained from all participating patients.

Exclusion criteria were as follows: (1) individuals who had taken proton pump inhibitors, H2 receptor antagonists, or other antacids, probiotics, mucosal protective agents, or antibiotics within the preceding 4 weeks (as all these medications directly impact the gastric mucosa flora); (2) a history of gastric adenoma, gastric cancer, or mucosa-associated lymphoid tissue lymphoma (all manifesting as alterations in the physiological structure of the gastric mucosa); (3) individuals who had undergone gastrectomy (as most of these patients have a history of advanced gastric cancer, leading to changes in the normal physiological structure of the stomach); and (4) patients who had undergone *H. pylori* eradication but were again found to be *H. pylori* positive [14].

Patients with upper gastrointestinal symptoms, diagnosed through gastroscopy and pathology as CG, and tested for *H. pylori* were included in this study. CG patients participating in the study underwent the ^13^C urea breath test (UBT), rapid urease test (RUT), and pathological histology staining of the gastric mucosa. *H. pylori* infection is defined as a positive result for pathological histology of the gastric mucosa, coupled with a positive outcome in either of the following two tests: (1) a UBT positive result exhibiting delta over baseline greater than 4.0% or (2) a positive RUT result. Furthermore, according to prior research on the gastric microbiome, samples with less than 1% relative abundance of *H. pylori* were excluded from the analysis to ensure higher representativeness [15].


**Informed consent:** Informed consent has been obtained from all individuals included in this study.
**Ethical approval:** The research related to human use has been complied with all the relevant national regulations, institutional policies and in accordance with the tenets of the Helsinki Declaration, and has been approved by the Medical Ethics Review Committee of the Affiliated Hospital of Youjiang Medical University for Nationalities (approval number: YYFY-LL-2017-01).

### 2.2 Animals

Twenty-four male C57BL/6 mice (20–25 g) aged 6–8 weeks were purchased from Sipeifu Biotechnology Co., Ltd. (Beijing, China). These animals were kept in a specific pathogen-free condition with a 12-h light/dark cycle, a room temperature of 22 ± 2°C, humidity between 50 and 60%, and free access to both food and drinking water. Approval for all experimental procedures was obtained from the Medical Ethics Review Committee of the Affiliated Hospital of Youjiang Medical University for Nationalities (approval number: YYFY-LL-2017-01), adhering to the National Research Council’s Guide for the Care and Use of Laboratory Animals.


**Ethical approval:** The research related to animal use has been complied with all the relevant national regulations and institutional policies for the care and use of animals and has been approved by the Medical Ethics Review Committee of Youjiang Medical University for Nationalities (approval number: YYFY-LL-2017-01).

### 2.3 Collection of sequencing sample

The gastric body stands as the prevalent site for autoimmune gastritis, whereas the gastric sinus represents the most frequent site of atrophic gastritis. In this investigation, a specimen was obtained from the gastric body, and the other specimen was acquired from the gastric antrum, and the two samples underwent combined sequencing. The specific procedural steps were as follows: one piece of gastric sinus mucosa and one piece of gastric body mucosa were extracted under gastroscopy utilizing disposable sterile biopsy forceps and then loaded into sterile sealed 5 ml lyophilized tubes containing 2 ml of sterile saline. Following labeling the tubes and recording the corresponding specimen information, the tubes were promptly placed in a thermos flask containing liquid nitrogen to arrest the intrinsic enzyme activity and oxidation. Subsequently, the tubes were transported to the laboratory of the Experimental Center of the Youjiang Medical University for Nationalities within 1 h for freezing and stored frozen at −80°C. These samples were later employed for high-throughput sequencing of 16S rRNA.

### 2.4 Sequencing process

Genomic DNA was extracted from 26 gastric mucosa samples utilizing a DNA extraction kit following the manufacturer’s protocols, and the purity and concentration of the DNA were assessed through 1.2% agarose gel electrophoresis. Employing the diluted genomic DNA as a template, PCR amplification of the target fragment (bacterial diversity identification region: 16S V3-V4 region) was conducted with specific primers designed based on the conservative regions in the sequence with high-fidelity enzymes. Post-electrophoretic detection, the PCR amplification products underwent purification with magnetic beads and served as templates for the second-round PCR. Subsequently, the second-round PCR amplification, electrophoresis, and purification were conducted. The final PCR products were quantified using the Quant-iT PicoGreen dsDNA Assay Kit and subjected to sequencing on the machine after passing the quality control. The PCR primers are detailed in Table 1.

**Table 1:** PCR primers

Sequencing	Primer	Primer sequence	Region
Bacteria	343F	5′-TACGGRAGGCAGCAG-3′	V3–V4
16S rRNA	798R	5′-AGGGTATCTAATCCT-3′	

### 2.5 Bioinformatics analysis

The Illumina Miseq sequencing platform has the ability to concurrently sequence 16S rRNA genes from multiple samples and has been extensively employed for the investigation of the evolution and diversity among microbial species. Following quality filtration, elimination of chimeric sequence and the final sequence were used for operational taxonomic unit (OTU) clustering. In this process, parameters with sequence similarity greater than or equal to 97% were grouped into one OTU unit, and all representative sequences were annotated against the database [16]. Various analyses were performed, including OTU clustering analysis, alpha diversity analysis, beta diversity analysis, community composition analysis, species difference analysis, and Phylogenetic Investigation of Communities by Reconstruction of Unobserved States (PICRUSt) functional prediction analysis.

### 2.6 Quantitative real-time PCR (qRT-PCR)

qPCRs were conducted using the FTC-3000TM Real-Time Quantitative Thermal Cycler (Funglyn, Shanghai, China). All qPCRs were performed with three replicates per DNA sample. Standard curves were established by serially diluting the plasmid of a pMD18-T vector containing the appropriate insert from 10^7^ to 10^12^ target gene copies per μl for each primer set. The standard curve was generated using linear regression of threshold cycle numbers (Ct) versus log copy numbers of targets.

The qPCRs were executed in 25 μl of reaction mixtures composed of 12.5 μl SRBR Premix Ex Taq™ (2×) (Takara, Japan), 1 μl (10 μM) of each forward and reverse primers (357F and 806R), 5 μl of template DNA, and sterilized deionized water. The reaction conditions were as follows: pre-denaturation at 95°C for 30 s, denaturation at 95°C for 10 s, annealing at 55°C for 30 s, extension at 72°C for 30 s (40 cycles), and a final extension at 72°C.

### 2.7 Establishing the model of *H. pylori* infection in mice

Subsequent to a 1-week acclimatization period, the mice were arbitrarily apportioned into two cohorts, each comprising 12 mice. The establishment of the animal model followed the patent of Dr. Yanqiang Huang (Patent No. ZL201910245354.5) [17]. Mice in the HP_Pos group underwent gavage with a clinically isolated *H. pylori* strain, diluted to a suspension of 1 × 10^9^ CFU/ml. This administration involved a volume of 0.5 ml per rodent, administered once every 2 days for a total of five instances. Fasting and water deprivation for 12 h before gavage, and fasting and water deprivation for 4 h after gavage. In the meantime, the mice in the HP_Neg group received an equivalent volume of physiological saline.

Extensive pre-experimental studies conducted by our team confirmed that the mouse *H. pylori* model, constructed as per the present invention, achieves a success rate of 90% within 3 months [17]. At the 6- and 12-week intervals following the final gavage, six mice from each group were randomly selected for sacrifice through cervical dislocation, respectively. Gastric tissues of the mice were expeditiously harvested for routine hematoxylin–eosin (H&E) staining and pathological scrutiny. Moreover, gastric juice was collected and underwent volumetric quantification and concentrations of acid were determined through titration.

### 2.8 H&E staining

The stomachs of the mice underwent immersion in a 10% neutral buffered formalin solution for fixation. Subsequent to this procedure, a strip extending from the gastric sinus to the gastric body was collected and embedded in paraffin. Then, the strips were sectioned into 4 μm pieces, followed by routine H&E staining. The images were captured utilizing a microscope.

### 2.9 Enzyme-linked immunosorbent assay (ELISA)

The determination of gastric malondialdehyde (MDA) concentration was carried out employing the thiobarbituric acid (TBA) reactive substances assay kit (Solarbio, Beijing). The underlying principle involves the reaction between one molecule of MDA and two molecules of TBA, yielding a red MDA-TBA complex under high-temperature (90–100°C) and acidic conditions, which can be quantitated using a spectrophotometer at 532 nm. The assay procedures were executed as described. The concentration of MDA was expressed in terms of nmol/mg protein.

### 2.10 Statistical analysis

Alpha diversity analysis was conducted utilizing the Mann–Whitney *U*-test. For the assessment of beta diversity, two- and three-dimensional principal co-ordinate analysis (PCoA) plots were generated based on the branch length of the phylogenetic tree shared between the groups. The Wilcoxon rank-sum test was employed to carry out the analysis of distinctions between the two groups of species. Independent sample *t*-tests were employed for the inter-group comparison of measurement data, and χ^2^ tests were applied for inter-group comparisons of counting data using SPSS for Windows, version 20. All *P*-values were bilateral, and a *P*-value of < 0.05 was considered statistically significant.

## 3 Results

### 3.1 Characteristics of the patients

Twelve *H. pylori*-positive CG patients and 14 *H. pylori*-negative CG patients were included in this study. The age of the enrolled patients ranged from 37 to 84 years old, and there was no noteworthy distinction in terms of age and gender between the two groups (Table 2).

**Table 2:** Comparison of baseline characteristics (mean  ±  SD)

Characteristics	HP_Pos group (*n* = 12)	HP_Neg group (*n* = 14)	*t*/χ^2^ value	*P* value
Age (years)	49.33 ± 16.44	53.00 ± 11.27	0.671	0.508
**Gender (** ***n*** **, %)**				
Male	5 (41.67)	7 (50)	0.154	0.695
Female	7 (58.33)	7 (50)		

HP_Pos: *H. pylori* positive; HP_Neg: *H. pylori* negative.

### 3.2 OTU clustering analysis

**Figure 1:**
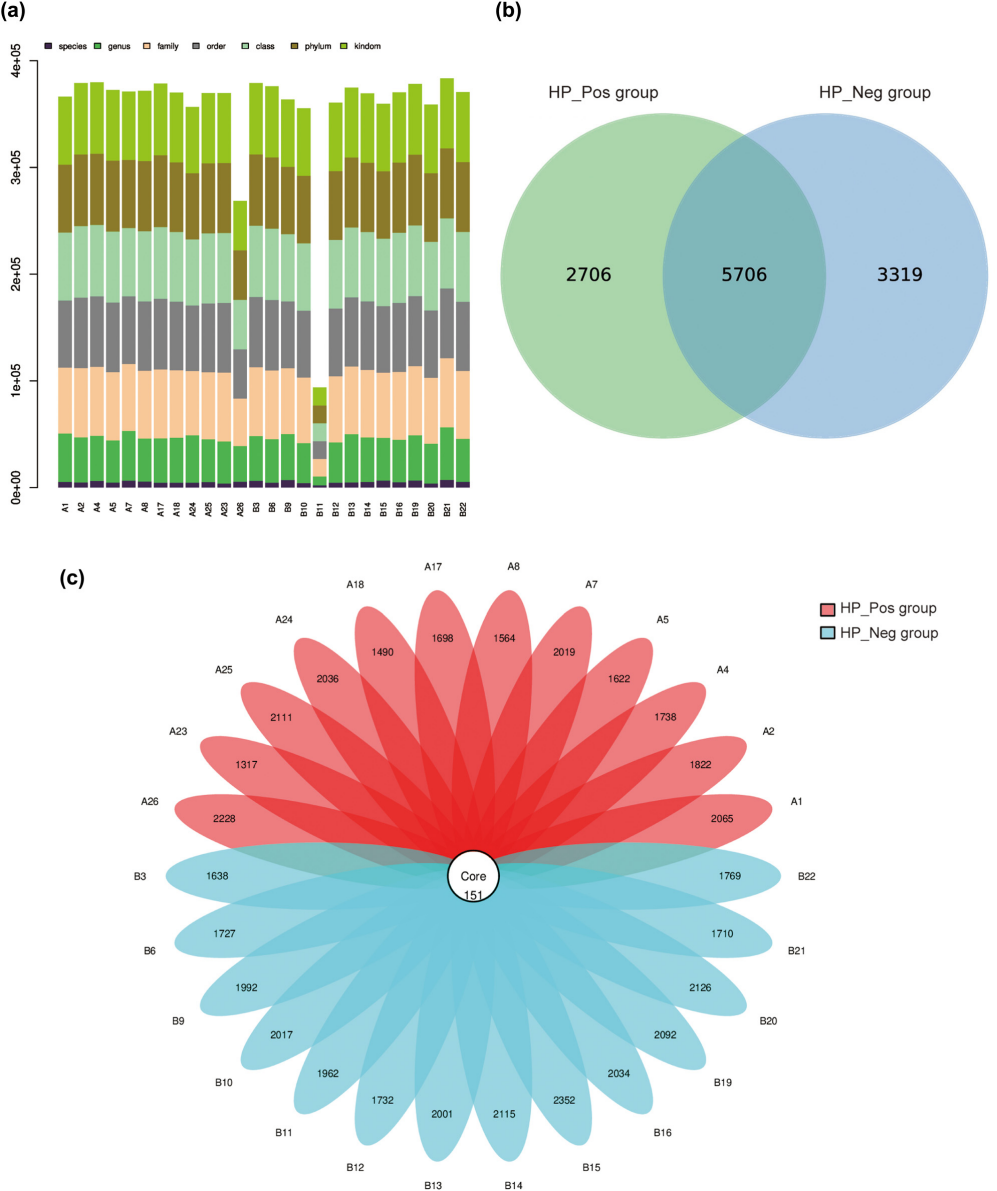
OTU clustering analysis of gastric mucosa microflora. (a) OTU level barplot; (b) Venn diagram demonstrates the shared and unique OTUs in both groups; and (c) the flower petal diagram of OTUs in both groups.

A total of 131,981 valid sequences were acquired from 26 samples utilizing Illumina high-throughput sequencing technology. Tags were annotated at each taxonomic level, that is, the aggregate count of tags corresponding to OTUs at each taxonomic level of the kingdom, phylum, class, order, family, genus, and species were summarized to generate an OTU level barplot illustrating the annotation proportion of each sample (Figure 1a).

OTU clustering, executed with a 97% similarity threshold, yielded a total of 11,731 OTUs. The Venn diagram generated by R software revealed that the HP_Pos group possessed 8,412 OTUs, while the HP_Neg group obtained 9,025 OTUs. The un-overlapped portion denoted the number of OTUs unique to each group, with the HP_Neg group exhibiting 613 more unique OTUs than the HP_Pos group. Notably, 5,706 OTUs were shared by the two groups, signifying a considerable degree of similarity in the bacterial structure between the two groups (Figure 1b).

Based on the results of the OTU clustering analysis, sample optimization of each group was conducted, and the overlap between each sample was displayed in the form of petals to elucidate the quantity of OTUs per sample and the cumulative OTUs count across all samples. The flower petal diagram conveyed that the overall number of OTUs in all samples was 151 (Figure 1c).

### 3.3 Alpha diversity analysis

**Figure 2:**
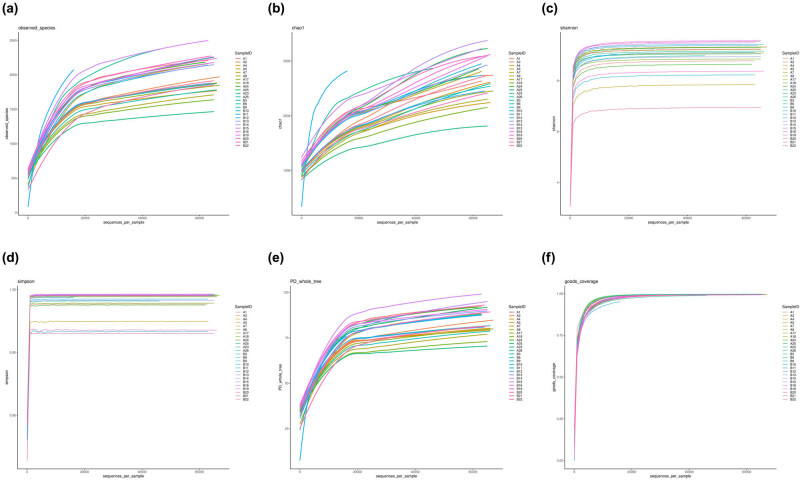
Alpha diversity analysis of gastric mucosa microflora. (a) Observed species curve; (b) Chao1 curve; (c) Shannon curve; (d) Simpson curve; (e) PD dilution curve; and (f) good coverage curve.

Alpha diversity analysis was employed to assess the abundance and diversity of microorganisms in the samples. Various indices, including Observed_species (Figure 2a), Chao1 (Figure 2b), Shannon (Figure 2c), Simpson (Figure 2d), PD_whole_tree (Figure 2e), and goods_coverage (Figure 2f), were employed to construct the corresponding exponential sparse curves at the OTU level for all samples. These curves served to evaluate the adequacy and rationality of the sequencing amount of samples and reflect the species richness of the samples. The dilution curve demonstrated a tendency to plateau, indicating that the sequencing volume saturation of the samples in both groups was sufficient, and the bacterial species richness, species coverage, and sample uniformity were reasonable. The specaccum species accumulation curve (Figure S1a), generated by R software, displayed a diminishing increase rate, leveling off with the expansion of the sequencing sample size. This observation suggested that the sample size covered most bacterial communities and could reflect the species richness. The gentle ascent of the curve implied that species in this environment would not substantially increase with the increase in sample size, indicating that the sampling was sufficient. The rank abundance curve (Figure S1b) further revealed that the majority of samples in the two groups displayed wide and flat horizontal curves with a gentle broken line, indicating that the species richness and evenness of most samples were reasonable, and the sequencing quantity could cover the majority of bacterial species, which was sufficient for subsequent analyses.

Based on the calculation of the diversity indices across different samples at unified depth, the violin plots of the diversity indices for different groups were generated to depict the distribution of the above six diversity indices in the two groups and to visually assess the degree of sample dispersion and the high and low differences of the indices between the two groups (Figure S2). According to statistical analysis, although there were differences in the alpha diversity index between the groups, the differences were not statistically significant, reflecting that there was no substantial distinction in the community richness and diversity between the samples of the HP_Pos group and the HP_Neg group (Table 3).

**Table 3:** Comparative analysis of α diversity index between the HP_Pos group and the HP_Neg group

α diversity index	HP_Pos group	HP_Neg group	*P* value
Observed_species	1555.87 ± 181.16	1674.04 ± 200.41	0.23
Chao1	1793.42 ± 274.637	1951.36 ± 312.69	0.43
Shannon	8.913 ± 0.43	8.93 ± 0.72	0.27
Simpson	0.99 ± 0.01	0.99 ± 0.01	0.40
PD_whole_tree	72.39 ± 5.38	75.66 ± 5.12	0.16
Goods_coverage	0.98 ± 0.01	0.978 ± 0.01	0.40

HP_Pos: *H. pylori* positive; HP_Neg: *H. pylori* negative.

### 3.4 Beta diversity analysis

**Figure 3:**
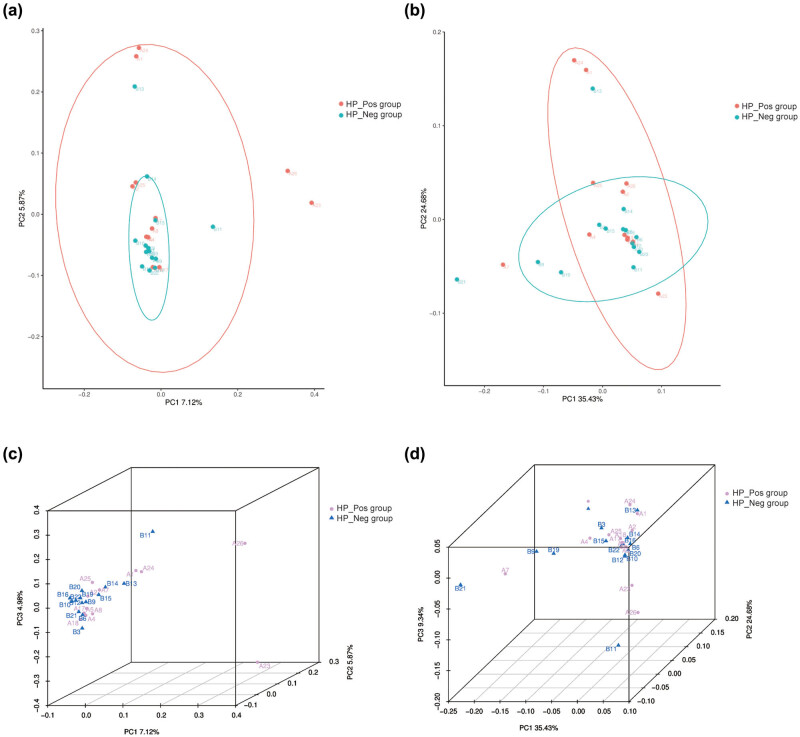
Beta diversity analysis of gastric mucosa microflora. PCoA plot (2D features) with (a) unweighted uniFrac and (b) weighted uniFrac distance metrics; PCoA plot (3D features) with (c) unweighted uniFrac and (d) weighted uniFrac distance metrics.

Beta diversity serves as an indicator of the diversity disparity among different samples and gauges the similarity in microbial composition between individuals. Consequently, the PCoA 2D (Figure 3a and b) and PCoA 3D (Figure 3c and d) were employed to investigate the distinctions in β diversity between the samples of the two groups. The outcomes indicated that neither unweighted uniFrac nor weighted uniFrac PCoA could effectively distinguish the gastric mucosal bacterial community composition between the HP_Pos group and the HP_Neg group; there was clustering and overlap, with no statistical difference in β diversity (*P* > 0.05).

### 3.5 Distribution of community structure

According to the taxonomic level of phylum, class, order, family, genus, and species, the top 15 abundance ranking (TOP15) species were plotted as histograms. The *x*-axis represented the sample, presenting the proportion of each species in each sample. Additionally, heatmaps were generated based on the abundance of species in the TOP15 species of each sample at various taxonomic levels by clustering taxonomic information and inter-sample variation to visually compare the species abundance at different taxonomic levels in each sample.

**Figure 4:**
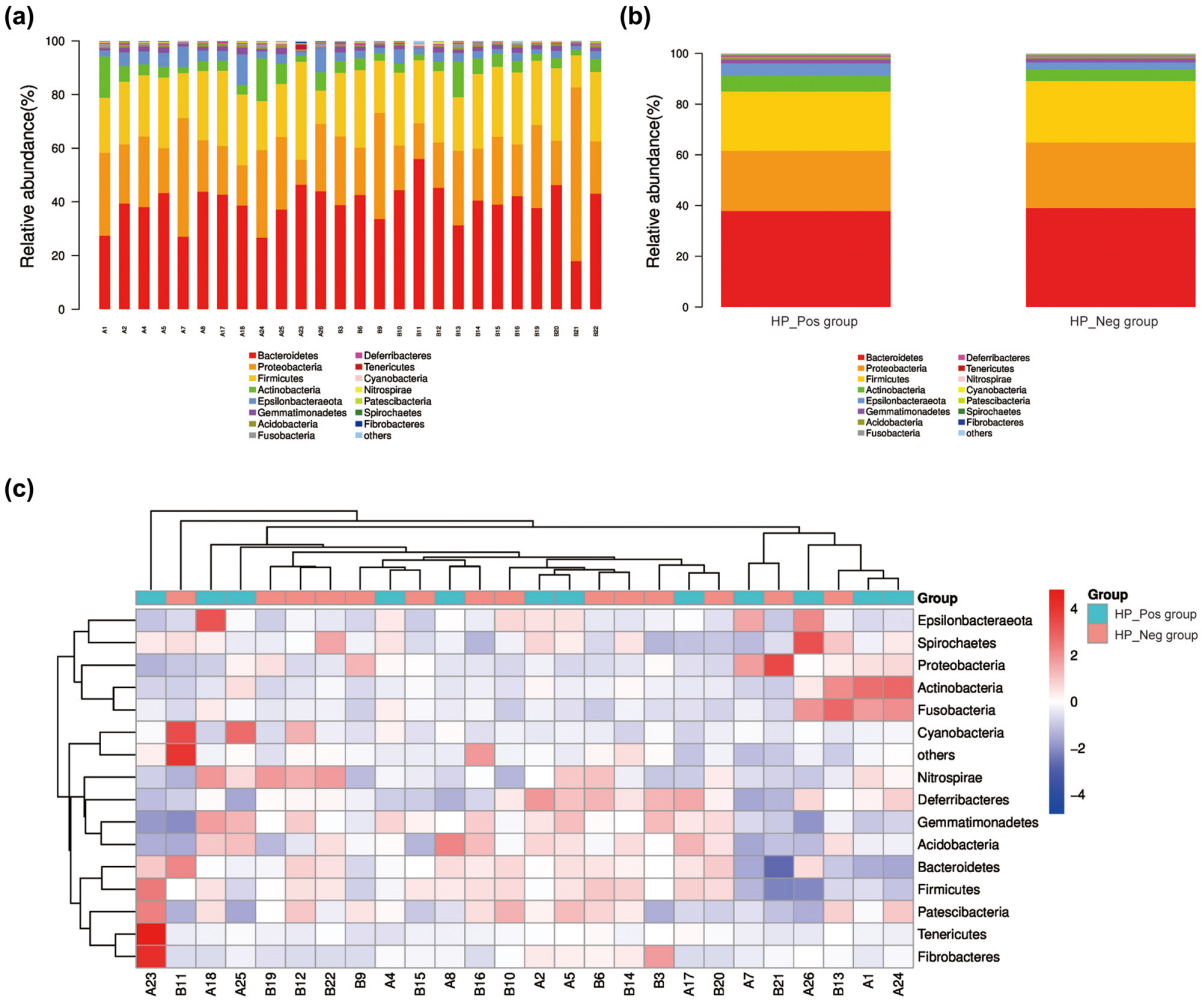
Species distribution at the phylum level. (a) Histogram of the relative abundance of bacteria in gastric mucosa of all samples; (b) histogram of the relative abundance of bacteria in gastric mucosa of two groups; and (c) the TOP15 phylum level abundance cluster diagram of all samples.

The community composition of all samples and samples between the groups were analyzed at the phylum level (Figure 4). The results revealed that the gastric mucosal flora in both two groups was primarily distributed in nearly 15 phyla. Among them, Bacteroidetes (37.92%) exhibited the highest bacterial abundance in the stomach of *H. pylori*-positive CG patients, followed by Proteobacteria (23.78%), Firmicutes (23.46%), Actinobacteria (6.20%), and Epsilonbacteraeota (4.95%). Similarly, the TOP5 dominant phyla in the intragastric flora of *H. pylori*-negative CG patients were consistent with those of *H. pylori*-positive CG patients, albeit with varying proportions, which were Bacteroidetes (39.08%), Proteobacteria (25.96%), Firmicutes (24.35%), Actinobacteria (4.55%), and Epsilonbacteraeota (2.83%), respectively. Regarding inter-group comparisons, the proportions of Bacteroidetes (*P* = 0.95), Proteobacteria (*P* = 0.80), and Firmicutes (*P* = 0.99) in the HP_Neg group were higher than those in the HP_Pos group, with no significant difference; while the proportions of Actinobacteria (*P* = 0.24) and Epsilonbacteraeota (*P* < 0.05) in the HP_Neg group were lower than those in the HP_Pos group.

**Figure 5:**
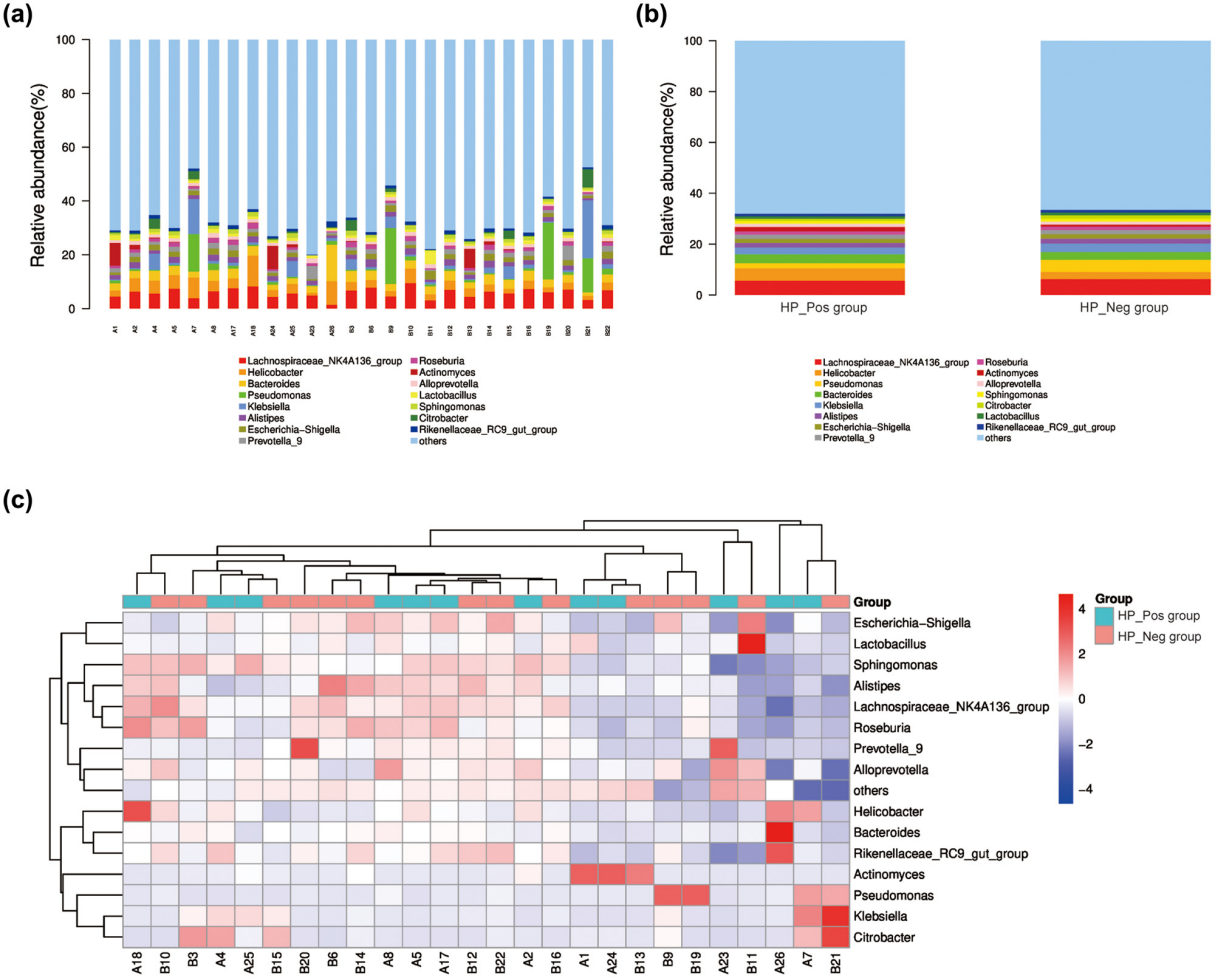
Species distribution at the genus level. (a) Histogram of the relative abundance of bacteria in gastric mucosa of all samples; (b) histogram of the relative abundance of bacteria in gastric mucosa of two groups; and (c) the TOP15 genus level abundance cluster diagram of all samples.

**Figure 6:**
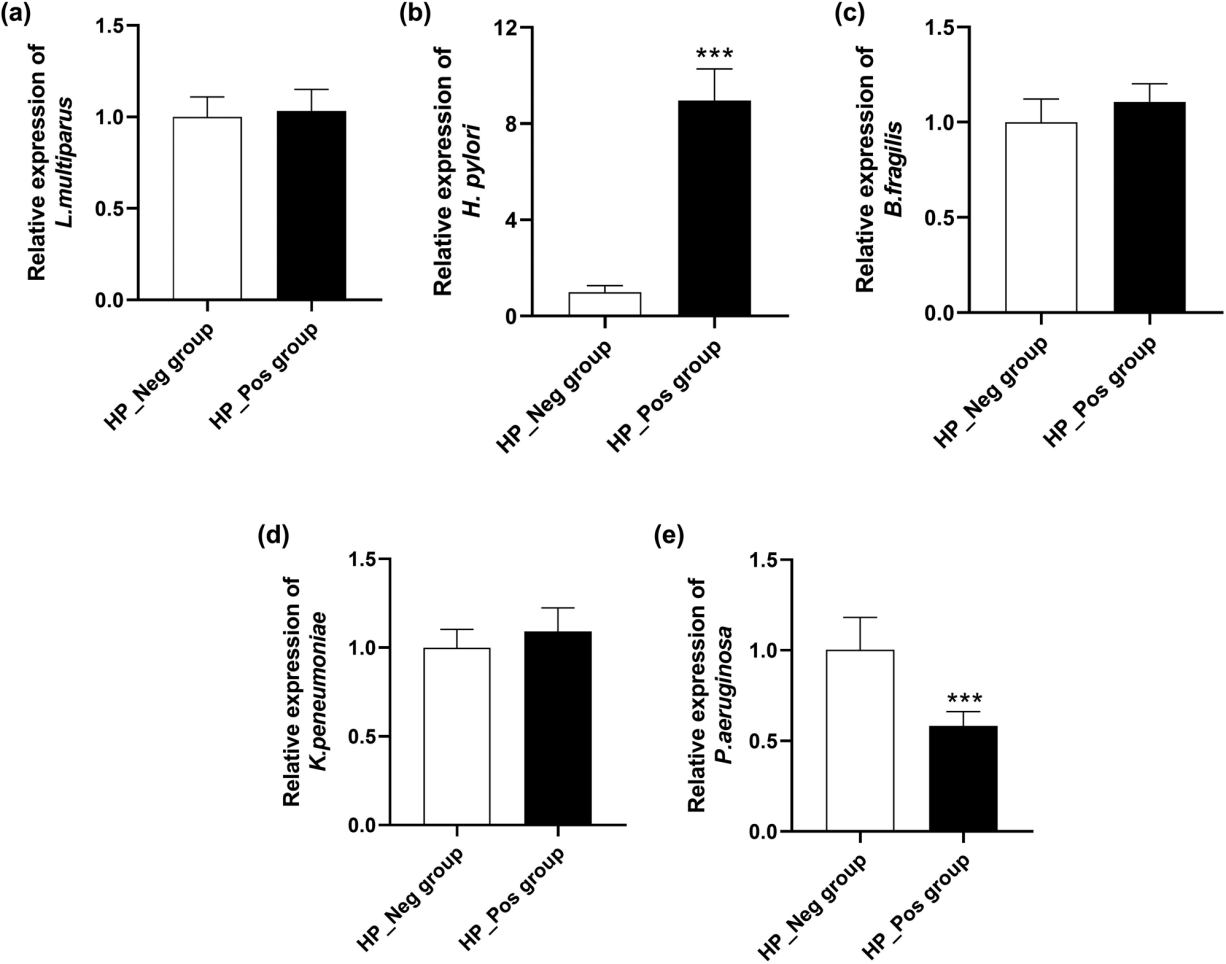
The expression of the TOP5 genera in the samples of both two groups detected via PCR analysis. (a) *L. multiparus*; (b) *H. pylori*; (c) *B. fragilis*; (d) *K. peneumoniae*; and (e) *P. aeruginosa*.

At the genus level, there was no significant difference in the composition map of gastric flora between the two groups (Figure 5). In the HP_Pos group, *Lachnospiraceae_NK4A136_group* exhibited the highest abundance, accounting for about 17.63%, followed by *Helicobacter* (15.24%), *Bacteroides* (11.22%), *Klebsiella* (8.27%), and *Pseudomonas* (6.12%), respectively. Moreover, there were 10 genera constituted more than 1%, including *Alistipes* and *Escherichia-shigella*. The results of community distribution in the HP_Neg group revealed that *Lachnospiraceae_NK4A136_group* was also the most abundant genus, accounting for a high percentage of 18.73%, followed by *Pseudomonas* (14.30%), *Klebsiella* (9.97%), *Bacteroides* (9.17%), and *Helicobacter* (8.13%). The proportion of dominant bacteria exhibited variance between the two groups. In the stomach of *H. pylori*-positive CG patients, *Helicobacter* emerged as the second dominant bacteria (*P* < 0.05), and the proportion of *Pseudomonas* was remarkably reduced compared to *H. pylori*-negative CG patients (*P* < 0.05). The content of the representative strains, namely *L. multiparus*, *H. pylori*, *B. fragilis*, *K. peneumoniae*, and *P. aeruginosa*, belonging to the TOP5 genus in the samples of both groups was further verified through qRT-PCR, and the obtained results were in agreement with the aforementioned findings (Figure 6).

The relative abundance of other taxonomic levels (class, order, family, and species) was depicted in histograms and heatmap in Figure S3.

### 3.6 Differences in microbiome compositions between the groups

Multivariate statistical analysis of microbial variables was employed to identify species with significant differences between the two groups. As depicted in Table 4, at the *T*-test level, there were 108 differential OTUs, 1 differential phylum, and 23 differential genera between the two groups. At the Wilcoxon rank-sum test level, the number of differential OTUs, differential phylum, and differential genera was 322, 2, and 46, respectively. Furthermore, the linear discriminant analysis effect size analysis was utilized to identify the potential differences in the abundance of the various bacterial taxa between the two groups (all had LDA scores >3.0 and *P* <  0.05, Figure S4).

**Table 4:** The number of species differences between the HP_Pos group and the HP_Neg group at different taxonomic levels

Method	Out	Phylum	Class	Order	Family	Genus	Species
*T*_test	108	1	4	4	8	23	15
Wilcoxon	322	2	5	11	14	46	31

**Figure 7:**
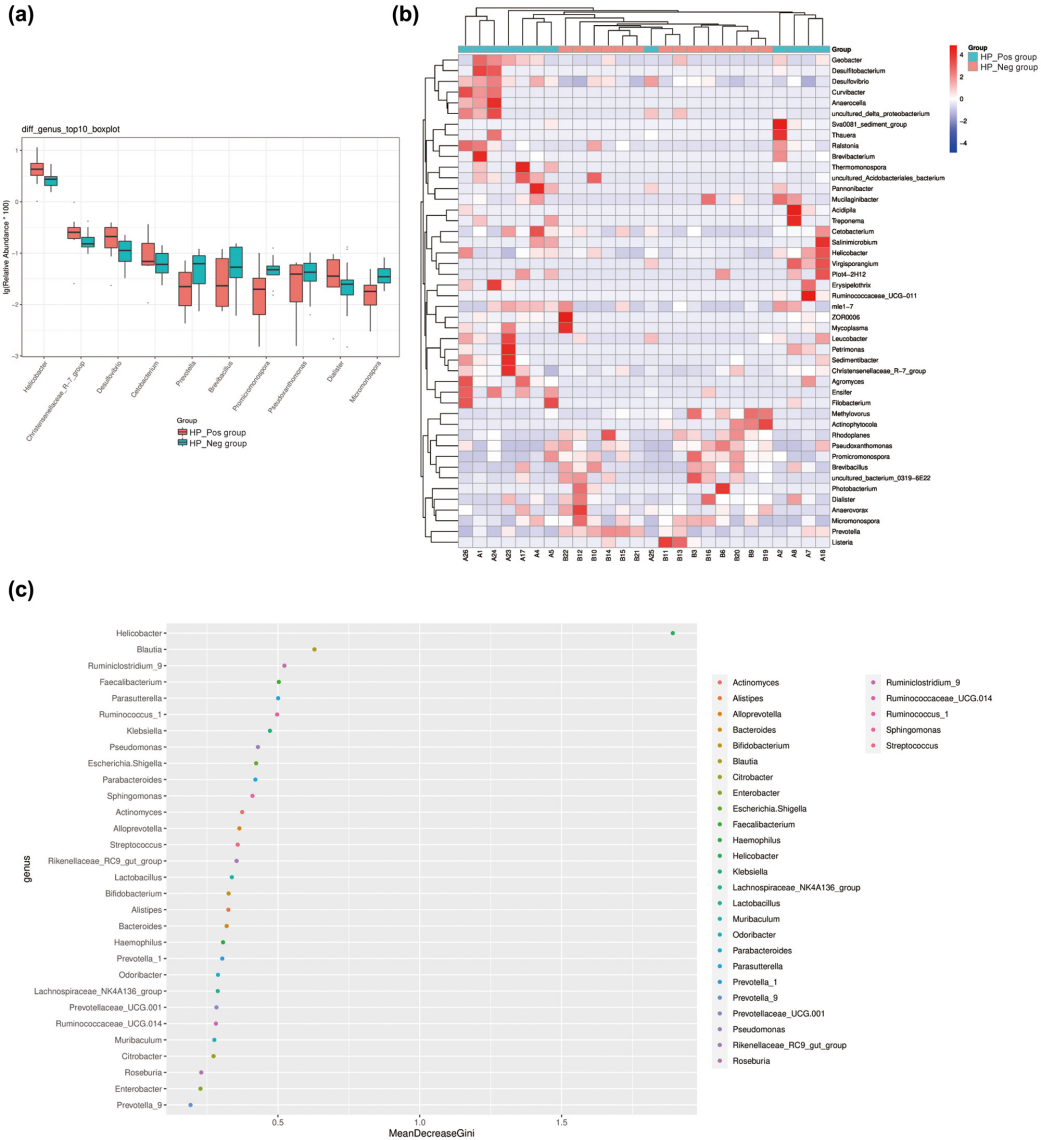
Multivariate statistical analysis of microbial variables. (a) TOP10 boxplot and (b) TOP10 heatmap of different species abundance (at genus level) in gastric mucosa of two groups. (c) Important species at the genus level were analyzed by random Forest analysis.

Significant differences in the genera of the TOP10 species with diverse abundance were observed between the groups, as analyzed by the Wilcoxon rank-sum test (*P* < 0.05, Table 5, Figure 7a). Specifically, the abundances of *Helicobacter*, *Christensenellaceae_R-7_group*, *Desulfovibrio*, *Cetobacterium*, *Prevotella*, *Brevibacillus*, *Promicromonospor*, *Pseudoxanthomonas*, *Dialister*, and *Micromonospora* differed significantly between the HP_Pos group and the HP_Neg group. Furthermore, the results of the heatmap analysis based on the relative abundance of species differing at the genus level revealed a discriminatory abundance at the genus level between the two groups (Figure 7b). The HP_Pos group exhibited higher abundances in *Geobacter*, *Esulfitobacterium*, *Desulfovibrio*, *Curvibacter*, *Anaerocella*, *Uncultured delta proteobacteriurn*, *Sva0081_sedirment_group*, *Thauera*, *Ralstonia*, *Brevibacteriurn*, *Thermomonospora*, *Uncultured_Acidobacteriales_bacterium*, *Pannonibacter*, *Mucilaginibacter*, *Acidipila*, *Treponema*, *Cetobacterium*, *Salininicrobium*, *Helicobacter*, *Virgisporangium*, *Plot4-2H12*, *Ruminococcaceae_ucG-a11*, *Mle1-7*, *Leucobacter*, *Petrimonas*, *Sodimentibacter*, *Christensenellaceae_R-7_group*, *Agromyces*, *Ensifer*, and *Filobacterium*. In contrast, genera such as *Mathylovarus*, *Actinophyrtocola*, *Rhodoplanes*, *Pseudoxanthononas*, *Pranicromonospora*, *Brevibecillus*, *Uncultured_bacterium_0319_6E22*, *Photabacterium*, *Dialister*, *Anaerovorax*, and *Micromonospora* displayed higher abundances in the HP_Neg group.

**Table 5:** Wilcoxon statistical difference species

Taxon	HP_Pos group	HP_Neg group	*P* value
*Helicobacter*	0.04961	0.02754	0.02
*Christensenellaceae_R-7_group*	0.00305	0.00183	0.03
*Desulfovibrio*	0.00216	0.00123	0.03
*Cetobacterium*	0.00113	0.00031	0.01
*Prevotella*	0.00030	0.00060	0.04
*Brevibacillus*	0.00024	0.00065	0.02
*Promicromonospora*	0.00017	0.00045	0.01
*Pseudoxanthomonas*	0.00018	0.00043	0.02
*Dialister*	0.00019	0.00036	0.04
*Micromonospora*	0.00013	0.00031	0.03

Utilizing random forest analysis to identify the key components (OTUs or species) capable of distinguishing differences between two groups of samples is an effective approach for classification and prediction. In this study, an analysis of the TOP30 genera in terms of relative abundance, coupled with the plotting of species importance points using a random forest model, revealed *Helicobacter* as the representative genus capable of stably distinguishing between the two groups of samples, indicating that it was a crucial measure (Figure 7c).

### 3.7 PICRUSt functional prediction analysis

**Figure 8:**
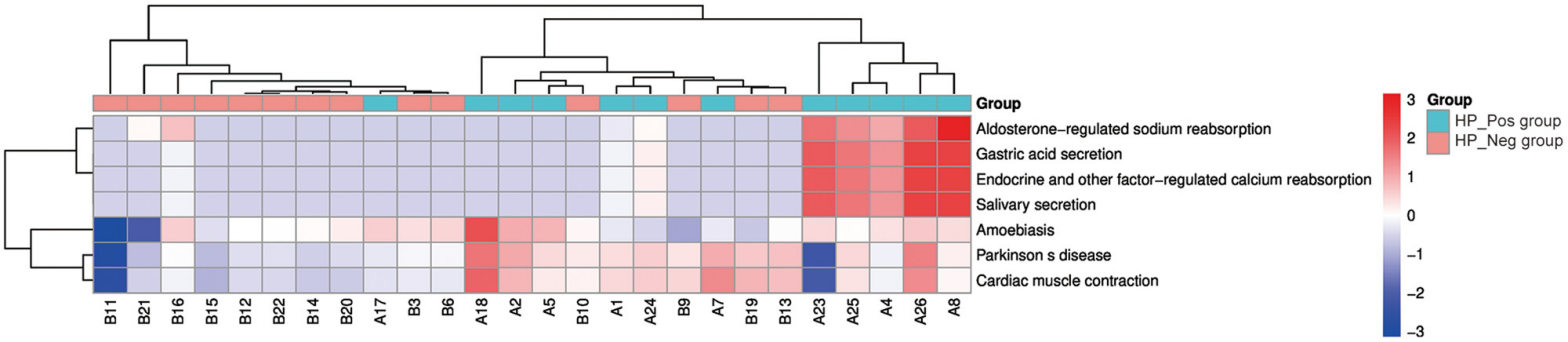
Heatmap graph of clustering of KEGG difference results.

Based on PICRUSt for predicting the metabolic pathways, the functional gene enrichment in Kyoto Encyclopedia of Genes and Genomes (KEGG) pathways at three different levels (KEGG_L1, KEGG_L2, and KEGG_L3) was obtained. The observed high expression of *H. pylori* at these three levels in the two groups of samples suggested that *H. pylori* might exert different weights on different functions (Figures S5–S7). According to the Wilcoxon test, there was no significant difference found between the two groups in the main pathway at the KEGG_L1 level; in the metabolic pathways at the KEGG_L2 level, only the circulatory system exhibited a significant difference between the two groups (*P* = 0.03); meanwhile, in the metabolic pathways at the KEGG_L3 level, the HP_Pos group and the HP_Neg group exhibited significant differences in seven functions, namely endocrine and other factor-regulated calcium reabsorption (*P* = 0.004), salivary secretion (*P* = 0.004), gastric acid secretion (*P* = 0.004), aldosterone-regulated sodium reabsorption (*P* = 0.014), amoebiasis (*P* = 0.027), Parkinson’s disease (*P* = 0.021), and cardiac muscle contraction (*P* = 0.027). According to the functional difference results of the Wilcoxon test, a heatmap diagram was drawn as depicted in Figure 8. The results revealed that five samples in the HP_Pos group exhibited higher expression in the functions of aldosterone-regulated sodium reabsorption, gastric acid secretion, endocrine and other factor-regulated calcium reabsorption, and salivary secretion. Additionally, the remaining samples in the HP_Pos group also demonstrated pronounced expressed in amoebiasis, Parkinson’s disease, and cardiac muscle contraction compared to those in the HP_Neg group.

### 3.8 The pathogenic mechanisms of *H. pylori* infection *in vivo* model

**Figure 9:**
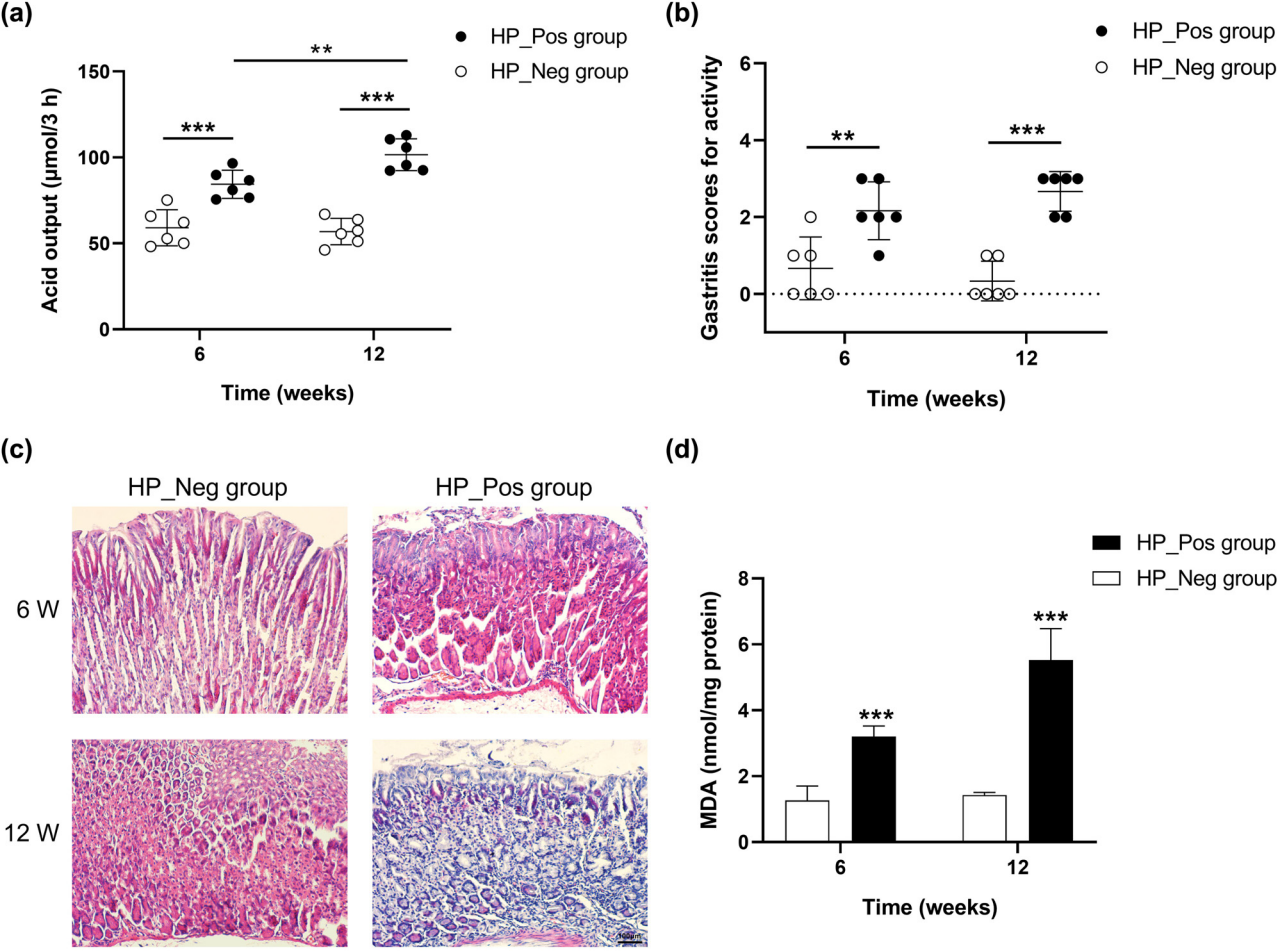
*In vivo* validation experiments for KEGG pathway. Alterations in (a) gastric acid secretion and (b) gastritis scores for activity observed at 6 and 12 weeks after inoculation in mice. (c) Histopathological changes in the gastric mucosa of mice detected by H&E staining. (d) Quantification of gastric MDA concentration determined via ELISA.

To gain a deeper comprehension of the impact of *H. pylori* infection on gastric acid secretion and the underlying pathogenic mechanisms of this bacterium, a mice model of *H. pylori* infection was established. The experimental cohort was designated as the HP_Pos group, wherein mice were subjected to *H. pylori* infection, while the HP_Neg group, serving as the control, received gastric lavage with an equivalent volume of physiological saline. Gastric acid secretion displayed a remarkable increase over the course of prolonged *H. pylori* infection (*P* < 0.01). Notably, in comparison to the HP_Neg group, the mice in the HP_Pos group consistently demonstrated significantly elevated levels of gastric acid secretion, observed distinctly at both 6 and 12 weeks (*P* < 0.001, Figure 9a). In addition, the recorded gastritis scores for activity were significantly increased in the HP_Pos group mice in comparison to those in the HP_Neg group at both the 6-week (*P* < 0.01) and 12-week (*P* < 0.001) intervals following inoculation (Figure 9b). The findings from H&E staining revealed pronounced infiltration of neutrophils and lymphocytes in mice infected with *H. pylori*, noticeable at both the 6- and 12-week intervals following inoculation. In contrast, minimal or absent occurrences of neutrophil and lymphocyte infiltrations were discernible in the HP_Neg groups (Figure 9c). Moreover, the determination of the MDA concentration through ELISA analysis revealed a congruence in trends with that of gastric acid secretion (Figure 9d).

## 4 Discussion

With the continuous advancement of high-throughput sequencing and other detection technologies, numerous investigations both domestically and internationally have illuminated the existence of a sophisticated microbial architecture and a distinctive microecological milieu in the human stomach. Nonetheless, the structure and functionality of the gastric flora, as well as its intricate relationship with human health and diseases, remain yet to be definitively elucidated. Consequently, in this study, the gastric mucosal tissues from *H. pylori*-positive and *H. pylori*-negative CG patients were collected, and the structural characteristics and differences in the gastric mucosal microbiota of these two patient cohorts were analyzed employing 16S rRNA high-throughput sequencing technology and PCR analysis. In addition, valuable insights into the impact of *H. pylori* infection on gastric acid secretion, along with its underlying pathogenic mechanisms, were gleaned through animal experiments. Our study has laid a robust foundation for examining the association between the composition of gastric microflora and the onset and progression of CG.

In the 16S gene variable regions, V3–V4 manifest the most extensive taxonomic coverage, diversity, reproducibility, and PCR amplification efficiency [18], rendering them the optimal choice for Illumina high-throughput sequencing. As per the outcomes emerging from the α-diversity analysis of OTUs, no notable dissimilarity was observed in the abundance and variety of gastric mucosal microbiota between *H. pylori*-positive and *H. pylori*-negative CG patients, indicating that the sampling in this study was adequate, with the sequencing volume achieving saturation sufficient to encompass the vast majority of species, the richness and homogeneity of the flora were high, and the samples obtained were ample for the subsequent analysis. In addition, the outcomes of the β-diversity analysis indicated a resemblance in the bacterial community structure of the gastric mucosa between the two aforementioned groups. Animal experimentation has demonstrated that the abundance and the community composition of the gastric mucosal flora in C57BL/6 mice, whether with acute or chronic *H. pylori* infection, experienced no noteworthy alterations [19]. These observations align with findings from human investigations. Both Khosravi et al. [20] and Coker et al. [21] discerned no noteworthy correlation between the composition of the human gastric flora and *H. pylori* infection, thereby lending support to the results obtained in our study.

In this investigation, it was discerned that the dominant bacteria in the stomach of CG patients, both with and without *H. pylori* infection, encompassed Bacteroidetes, Proteobacteria, Firmicutes, Actinobacteria, and Epsilonbacteraeota, but the proportion of these bacteria in the two groups was different. A study conducted by Cho et al. [22] similarly illustrated that the stomach microbes of both *H. pylori*-negative and *H. pylori*-positive individuals contained the same phylum, albeit in varying percentages of abundance. Although no significant disparity was observed in the α and β diversity of the gastric mucosal microflora between the two groups, the Wilcoxon statistical analysis unveiled noteworthy distinctions in the bacterial colonies at the levels of phylum, class, order, family, genus, and species, concurrently signifying that *H. pylori* infection may instigate disruption and disarray in the gastric microflora. Upon the colonization of *H. pylori*, an attenuation in the protective efficacy of the gastric mucosa ensued, giving rise to a sequence of pathological metamorphoses. This cascade culminated in the induction of disorder and dysbiosis in the gastric flora, accompanied by alterations in the gastric microenvironment [23]. Furthermore, the HP_Pos group exhibited markedly elevated proportions of Epsilonbacteraeota and Fusobacteria, distinctly surpassing their counterparts in the HP_Neg group. Epsilonbacteraeota, identified as one of the most prevalent phylum in patients with *H. pylori* infection, and the uniqueness of the flagellum of Epsilonbacteraeota may be one of the potential reasons for its significant divergence from other flora, which may be related to the mechanism of gastric mucosa inflammation in CG patients and could entail distinct metabolic functional pathways from the pathogenic *H. pylori* [24]. Fusobacteria, a gram-negative bacterium commonly found in the gastrointestinal tract, possesses invasive, adhesive, and inflammatory effects, playing a pivotal role in the development of colorectal cancer and its precancerous lesions by secreting substances that activate oncogenes and participate in the pro-carcinogenic pathway [25]. Hence, it is speculated that *H. pylori* infection might augment the invasion and metabolic capacity of Epsilonbacteraeota and Fusobacteria, which alter the inflammatory state of the gastric mucosa, and perhaps even accelerate the risk of chronic inflammation to tumorigenic transformation, the interrelationship among these three necessitate further comprehensive investigation.

Based on PICRUSt for predicting the metabolic pathways, the functional gene enrichment in KEGG pathways at three different levels was obtained. Upon scrutinizing the differential metabolic pathways and functional variations observed at the KEGG_L3 level heatmap between the two groups of samples, it is plausible to infer that *H. pylori* infection may exert a certain influence on endocrine function. Recent studies have compellingly suggested a modulator role for *H. pylori* in the endocrine system [26]. This participates in the secretion or dysregulation of specific functions, indicating a potential correlation with disorders associated with secretion dysregulation. Of particular interest is the elevated expression observed in the gastric acid secretion functionality in the HP_Pos group, evoking our keen interest in exploring this phenomenon further. Subsequently, we proceeded to establish a murine model of *H. pylori* infection, which yielded findings congruent with the sequencing results. Notably, a positive correlation surfaced between gastric acid secretion and the gastritis scores for activity; in other words, as the severity of gastritis increased, there was a corresponding augmentation in gastric acid secretion. In addition, histological analysis unveiled pronounced infiltration of neutrophils and lymphocytes in the mice infected with *H. pylori*. Prior investigations have elucidated that the pathogenic impact of *H. pylori* encompasses gastrointestinal tract colonization and substantial generation of reactive oxygen species (ROS) by the neutrophils congregating at the infection site [27,28]. Consequently, we conducted ELISA experiments to measure gastric MDA concentration, revealing a conspicuous elevation. This underscores the tendency of *H. pylori* to incite robust ROS production through the perpetual activation of neutrophils, thus accentuating gastric inflammation and amplifying gastric acid secretion, aligning with the existing literature reports.

In conclusion, this study revealed that while no significant differences were detected in the abundance, diversity, and structure of the bacterial flora in the gastric mucosa samples of CG patients between the two groups, there was a notable disparity in bacterial flora between the two groups at various classification levels, suggesting that *H. pylori* infection would instigate the disturbance of the gastric flora and imbalance of gastric microecological stability, leading to gastric dysfunction and a cascade of pathological processes through the related influence of other bacterial flora. Furthermore, *H. pylori* infection activates neutrophils continually attracted to the infected gastric mucosa, triggering substantial production of ROS. As a result, the inflammatory damage to the gastric mucosa becomes challenging to repair, and the infection remains chronic and inflammatory over an extended period. The limitations of this study primarily lie in the insufficient classification and grading of CG caused by *H. pylori* infection during endoscopy, and the number of cases included in this study remains relatively small. To comprehensively elucidate the interactions between *H. pylori* and other microbial communities within the stomach, it is imperative to augment the sample size in future investigations. In addition, the sequencing samples were exclusively obtained from the gastric body and the gastric sinus in this investigation. Nonetheless, as the gastric body and gastric sinus serve as primary sites of gastritis, they possess a certain capacity to reflect the overall microbial landscape within the stomach. Lastly, for the pathogenic flora that is co-involved in pathogenesis with *H. pylori*, future therapeutic approaches could focus on inhibiting its metabolic function to block its pathogenic pathway and diminish its value and pathogenic function.

## Supplementary Material

Supplementary Figure
