# Supplementary material for: Helicobacter pylori causes gastric dysbacteriosis in chronic gastritis patients
Source: Open Life Sci. 2024 Mar 28;19(1):20220839. doi: 10.1515/biol-2022-0839 (PMC10997148; doi:10.1515/biol-2022-0839)
Supplement: Supplementary Figure [file biol-2022-0839-sm.pdf]

Supplementary material

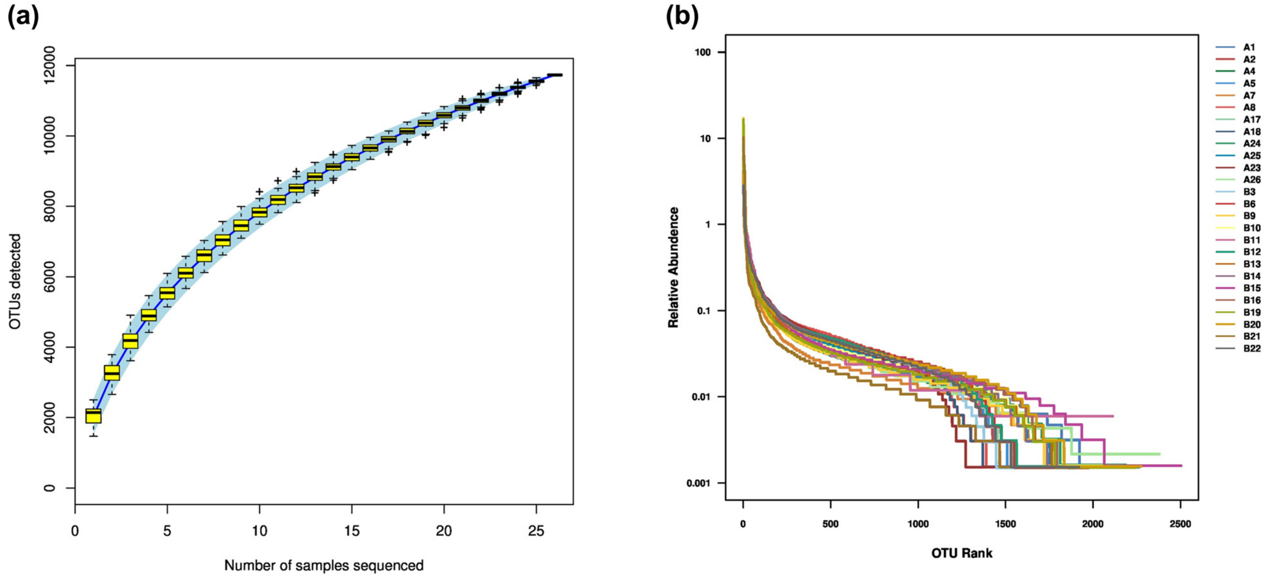

Figure S1: (a) Specaccum species accumulation curve; (b) Rank Abundance grade curve.

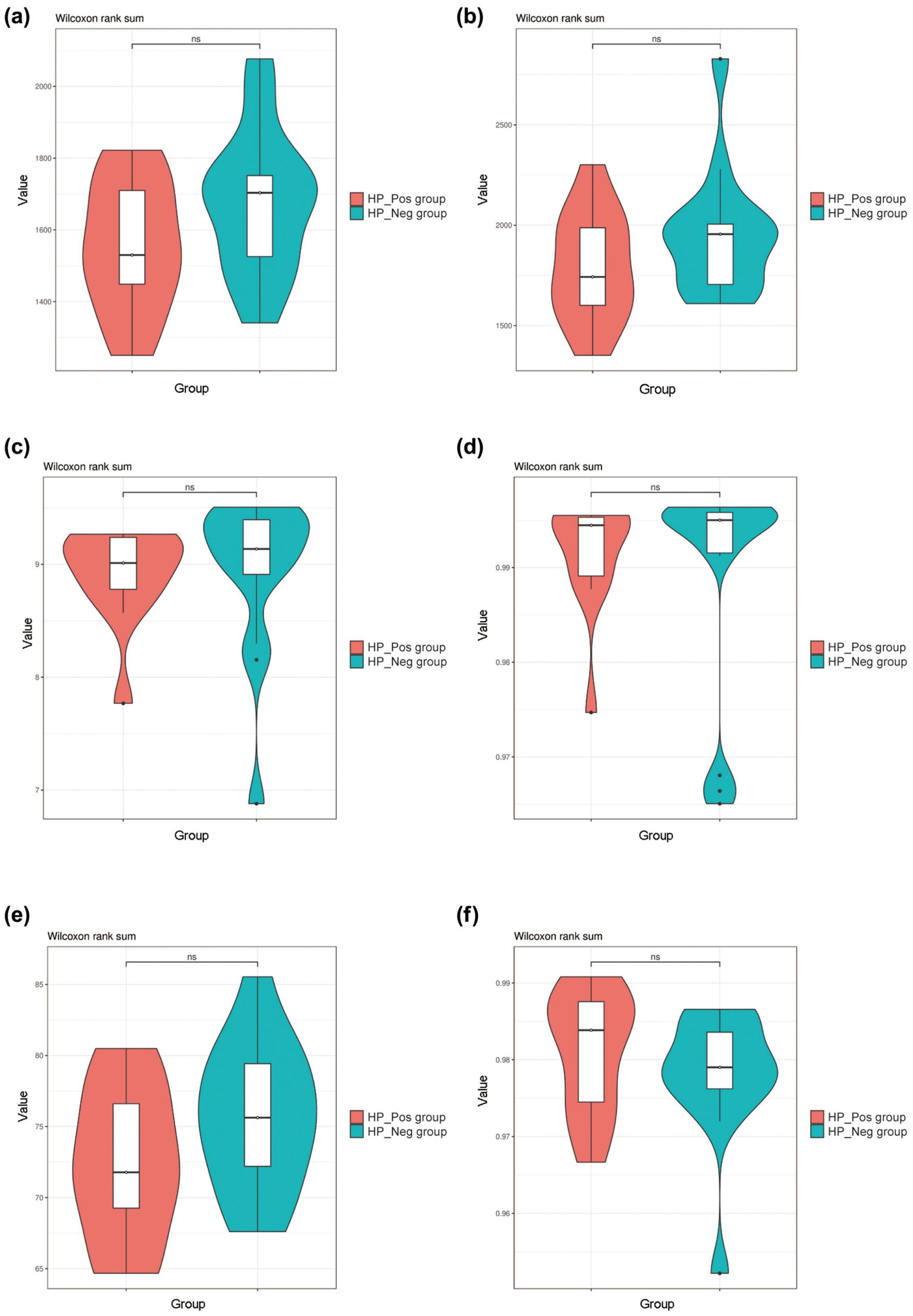

Figure S2: Alpha diversity-related violin analysis of gastric mucosa microflora. (a) Observed species curve; (b) Chao1 curve; (c) Shannon curve; (d) Simpson curve; (e) PD dilution curve; (f) Good coverage curve.

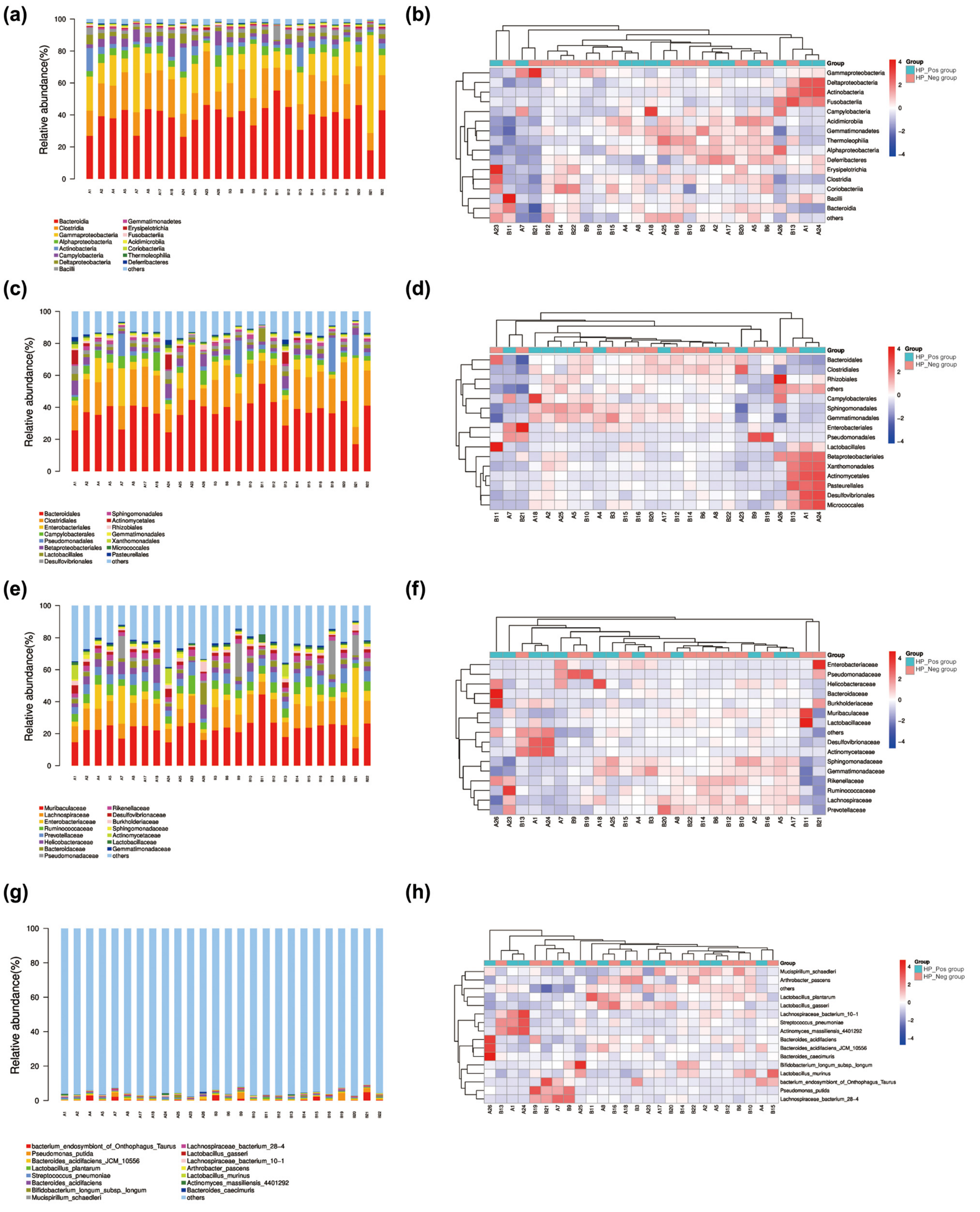

Figure S3: Relative abundance of class, order, family, and species levels. (a) Histogram of the relative abundance of bacteria in gastric mucosa and (b) the TOP15 phylum level abundance cluster diagram of all samples at the class level; (c) Histogram of the relative abundance of bacteria in gastric mucosa and (d) the TOP15 phylum level abundance cluster diagram of all samples at the order level; (e) Histogram of the relative abundance of bacteria in gastric mucosa and (f) the TOP15 phylum level abundance cluster diagram of all samples at the family level; (g) Histogram of the relative abundance of bacteria in gastric mucosa and (h) the TOP15 phylum level abundance cluster diagram of all samples at the species level.

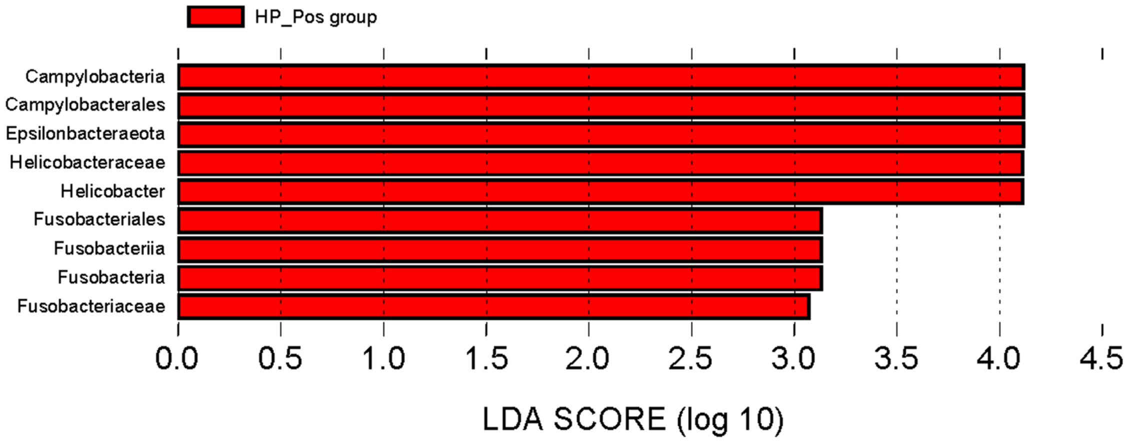

Figure S4: Association of specific microbiota taxa with the group of *H. pylori*-positive and *H. pylori*-negative by LEfSe (LDA score > 3.0, $P<0.05$).

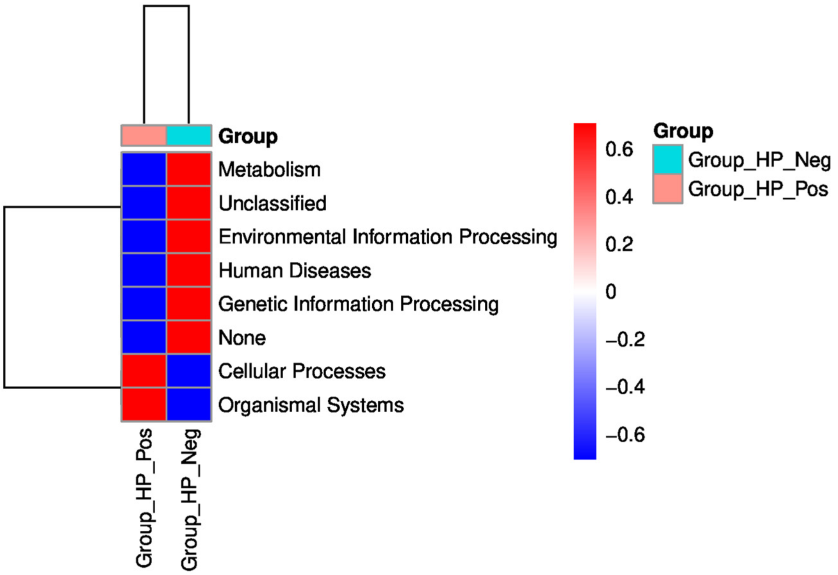

Figure S5: KEGG_ L1 horizontal heatmap.

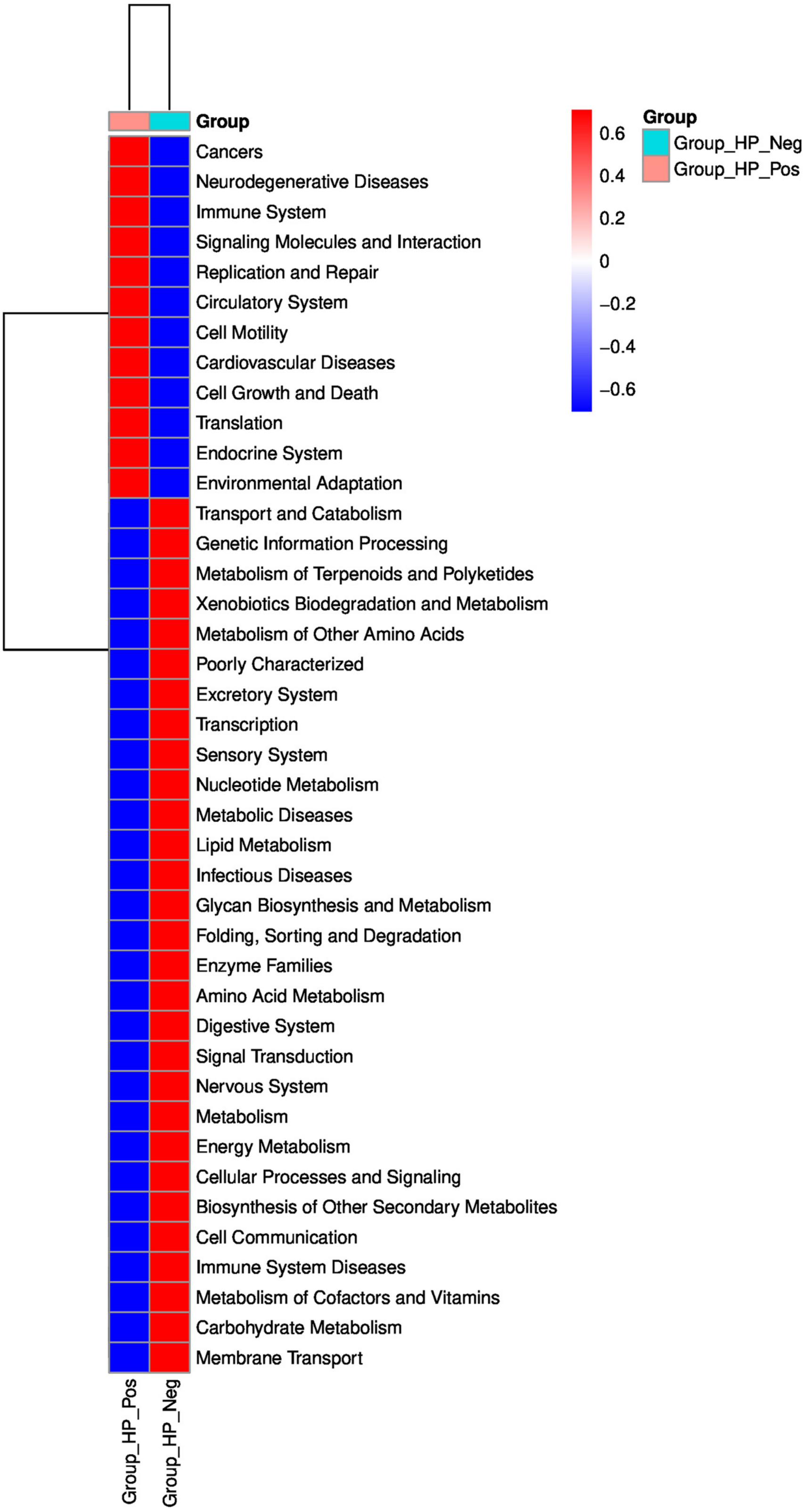

Figure S6: KEGG_ L2 horizontal heatmap.

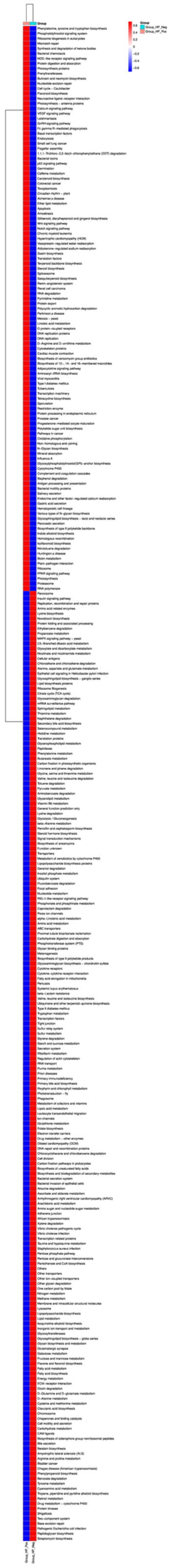

Figure S7: KEGG_ L3 horizontal heatmap.
